# Bridging the governance gap in health artificial intelligence: integrating nursing perspectives into the National Academy of Medicine Artificial Intelligence Code of Conduct

**DOI:** 10.1093/jamia/ocaf224

**Published:** 2026-02-25

**Authors:** Michael P Cary, Regina G Russell, Christina Silcox, Kay S Lytle, Lisa Soleymani Lehmann, Maia Hightower, Nicoleta Economou-Zavlanos, Vincent Guilamo-Ramos

**Affiliations:** Duke University School of Nursing, Durham, NC 27710, United States; Vanderbilt University School of Nursing, Nashville, TN 37203, United States; Duke-Margolis Institute for Health Policy, Washington, DC 20001, United States; Duke University Health System, Durham, NC 27710, United States; VA New England Healthcare System, Bedford, MA 01730, United States; Harvard Medical School, Boston, MA 02115, United States; Equality AI, Park City, UT 84060, United States; Department of Biostatistics & Bioinformatics, Duke University, Durham, NC 27710, United States; Johns Hopkins School of Nursing, Baltimore, MD 21205, United States

**Keywords:** artificial intelligence, delivery of healthcare, nurses

## Abstract

**Background and Approach:**

In 2025, the National Academy of Medicine released an Artificial Intelligence Code of Conduct (AICC). In this commentary, we examine how the AICC introduces governance mechanisms to oversee AI applications and how it can support the ethical development and responsible use of AI in healthcare, paying special attention to the role of nurses.

**Findings:**

One shortcoming of the AICC is its lack of explicit acknowledgment of nurses, which risks obscuring their indispensable role in the safe, equitable, and effective use of AI in healthcare. We offer practical steps for health leaders to operationalize the AICC.

**Conclusion:**

Implementation of the AICC can support robust AI governance in health systems, but nurse expertise must be incorporated. The AICC offers a framework into which nursing perspectives can be embedded to ensure that AI tools positively transform healthcare delivery and enhance the quality and equity of care.

## Introduction

Artificial intelligence (AI) has rapidly advanced over the past decade, transforming healthcare and offering significant potential for patient care, clinical decision-making, and health system efficiency. Despite these promising developments, reports of algorithmic bias and the worsening of existing inequities continue to raise concerns about fairness, transparency, accountability, and workforce impact.^1–3^ Extensive evidence demonstrates that clinical algorithms can amplify inequities if bias is not addressed through systematic governance, design review, and stakeholder inclusion.

Given the complex relationship between technological innovation and human-centered care, it is essential to tackle these issues. Therefore, the National Academy of Medicine (NAM) recently released an Artificial Intelligence Code of Conduct (AICC),^4^ providing a framework for ethically developing and responsibly using AI in healthcare delivery. Specifically, the AICC offers guidance on how health systems can operationalize core commitments such as advancing humanity, ensuring equity, engaging impacted individuals, improving workforce well-being, and fostering innovation and learning, thus influencing how various stakeholders build trust in health AI.

Notably missing from the AICC is an explicit acknowledgment and detailed integration of nursing roles and perspectives, despite nurses constituting the largest segment of the healthcare workforce and serving critical functions in patient care coordination and outcomes. This commentary focuses on the AICC’s implications for nursing as an underrepresented stakeholder group and aims to show how the explicit inclusion of the nursing perspective would help strengthen the AICC. While the AICC offers a broad ethical framework for the use of AI in healthcare, we focused on nursing without aiming to identify all potential gaps. We argue that to realize the full potential of the AICC, health systems must consider how to implement its recommendations, with critical steps being dedicating resources and building capacity to support nurse leadership participation in AI governance, development, and performance monitoring.

## Implementing strong AI governance frameworks in health systems

Health systems often implement AI without thorough governance, leading to inconsistent results, increased clinician burden, and potential patient harm.^5^ The introduction of the AICC framework addresses this issue by establishing strong governance mechanisms to oversee AI applications. It explicitly calls for cross-disciplinary oversight teams that include clinicians, data scientists, ethicists, and patient representatives to ensure transparency and accountability in AI deployment.^4^ By adopting such governance structures, health systems can ensure AI tools are not only clinically effective but also ethically sound and trusted by the clinicians who use them daily. Practical models already exist. For example, Duke Health’s algorithm oversight framework translates these ethical and quality principles into operational evaluation checkpoints across the lifecycle of AI tools.^6^ Additionally, health systems can lay a stronger foundation for responsible AI by requiring transparency from AI vendors about model training, validation, and limitations.^7^^,^^8^

## Embedding nursing expertise into health AI governance

Nurses represent the largest segment of the healthcare workforce and uniquely bridge patient care, operations, and clinical documentation. Nurses’ epistemology, grounded in relational care, empathy, and holistic assessment, positions them as ethical stewards who can inform algorithm design, feature selection, and labeling decisions. Their proximity to patients and real-time engagement with technology differentiate their role from that of other clinicians, making their inclusion critical for ensuring that AI tools fit workflows, promote safety, and advance equity.

Effective AI integration requires nurses to be informed participants rather than passive end-users.^9^ Including nurses on oversight teams is essential, given their frontline role in patient care and their practical insights on how AI tools impact clinical workflows and patient outcomes. Nurses often serve as the final safeguard in translating AI recommendations into clinical action. Their perspectives are vital for assessing AI tool usability, identifying unintended consequences, advocating for patients, and ensuring AI enhances rather than compromises care quality. Failing to explicitly recognize nurses perpetuates their underrepresentation in governance, policy, and implementation discussions. Because nurses are responsible for entering extensive clinical data into electronic health records, they offer essential insight into which data elements are most accurate, reliable, and contextually meaningful for AI development and evaluation. Embedding nurse perspectives early in model development can ensure that AI tools align with patient-centered values and minimize unintended biases. In addition, empowering nurses to participate in AI governance can improve tool usability, clinician satisfaction, and patient outcomes.^10^

Despite growing recognition of the importance of nursing input in AI design and oversight, persistent structural challenges constrain nurses’ ability to contribute meaningfully to governance efforts. Systemic barriers such as insufficient AI training opportunities, hinder meaningful nursing participation in AI governance. At present, there is no standardized curriculum in nursing that integrates key competencies (ie, AI ethics, data literacy, model interpretation, bias mitigation, and governance) into pre-licensure, graduate, or continuing education pathways. Thus, nursing involvement in AI efforts has been limited, resulting in the development and use of tools that often disrupt rather than improve clinical workflows.^11^^,^^12^ Overcoming the systemic barriers to nurses’ participation requires leadership commitment to resource allocation, mentorship programs, and interprofessional partnerships that empower nurse leaders in health AI strategy. Strengthening these supports will ensure that nursing expertise informs every stage of the AI lifecycle and advances responsible, patient-centered innovation in healthcare.

The AICC emphasizes workforce well-being and stakeholder engagement as critical priorities and explicitly advocates for the active involvement of frontline clinicians in AI design, development, and implementation. However, the lack of an explicit reference to nurses as a distinct professional group is a significant oversight. Although the framework uses broad terms like “clinicians” and “workforce,” this language risks obscuring the unique and essential role of nurses in ensuring safe, equitable, and effective AI use in healthcare. Advancing the AICC’s goals requires explicitly acknowledging nurses as key stakeholders who should be engaged throughout the AI lifecycle and empowered to influence AI systems to reflect patient care realities and uphold public trust.

One practical framework for operationalizing ethical commitments in healthcare is the Bias Elimination for Fair and Responsible AI in Healthcare (BE FAIR) framework.^10^ BE FAIR outlines how nurses are uniquely positioned to identify and mitigate bias throughout the AI lifecycle—from data collection and model design to evaluation and deployment. It designates nurses as equity stewards who help ensure that algorithms are developed and implemented in ways that promote fairness and transparency. For example, within BE FAIR, nurses play active roles in evaluating model inputs for representativeness, monitoring bias drift during deployment, and communicating fairness findings to clinical teams and patients. By applying BE FAIR, nurses can lead institutional efforts that embed equity, accountability, and patient-centered values into AI governance.

## From framework to practice: nursing’s role in implementing the AICC

In line with broader movements toward algorithmic accountability in healthcare, health systems and payors must take practical steps to operationalize the AICC framework successfully. A clear, step-by-step approach is necessary to embed the AICC principles into health system operations, ensuring safety, equity, and workforce engagement. Table 1 offers health leaders a useful guide to operationalizing the AICC framework, with an emphasis on the vital role of nurse leaders. For example, nurse-led initiatives in predictive modeling for sepsis and inpatient falls demonstrate improved model calibration by integrating nursing insights into patient monitoring, handoff communication, and documentation workflows.^13^ Such examples illustrate how nursing leadership can concretely improve both model performance and equity outcomes, but more evidence is needed.

**Table 1.** Leaders’ roadmap to implement the AICC: actions, timing, and evidence gaps.

Step	What?	How?	When?	Remaining evidence gaps
1. Build the Governance Infrastructure	Establish a structure to oversee AI use from start to finish.	Form a multidisciplinary AI governance committee. Explicitly include nursing leaders, physicians, data scientists, IT professionals, compliance professionals, and patient/family representatives. Define authority, scope, and decision-making rights. Use a charter, model registry, risk levels, and stage-gates (design → testing → implementation → monitoring).	Before selecting or piloting any AI tool	The AICC defines principles/commitments but does not prescribe concrete governance artifacts or role-level decision rights; these must be defined locally.
2. Engage Nursing Leadership Early	Ensure AI tools address real clinical needs and fit existing workflows.	Involve nurses in problem definition, vendor evaluation, and tool design. Conduct workflow mapping and pilot testing with bedside teams; perform human factors reviews; and commit to address alert burden and handoff liabilities flagged by nurses.	From initial project scoping through full implementation	Evidence shows potential harm to patients when frontline realities are not represented (eg, Epic Sepsis Model^14^ showed poor discrimination/calibration on external validation^15^); however, comparative studies quantifying the incremental benefit of nurse co-design are limited. Prospective, local evaluations are needed (eg, stepped-wedge, hybrid trials).
3. Build Foundational AI Literacy for Nurses and Other Frontline Clinicians	Enable participation and governance by nursing and allied health staff.	Develop targeted AI literacy programs (AI basics, bias detection, and interpreting outputs). Deliver via workshops, e-learning, and simulation training. AI literacy should progress across 3 levels: (1) foundational—understanding key AI concepts and ethical issues; (2) applied—interpreting model outputs and integrating them into clinical decisions; and (3) leadership—governing and evaluating AI tools. Integrating these competencies across pre-licensure, graduate, and continuing education ensures workforce readiness for AI-enabled care. Embed short courses; embed time micro-learning into the EHR; capture nurse-reported safety/usability signals as part of training outcomes.	Prior to pilot and as part of ongoing implementation (refresh at 30/90 days, then quarterly) During professional development	The AICC stresses workforce well-being and engagement but stops short of validated competency sets and instrumentation (eg, agreed metrics for alert burden, moral distress). Organizations should build local competency maps and pair them with model metrics.
4. Advance AI Skills in Nursing Education and Practice	Make AI competence a standard for future nursing practice.	Integrate AI ethics, governance, and data literacy into prelicensure, graduate, and CE curricula. Partner with academic programs to align training with workforce needs.	In parallel with workforce training; ongoing integration into education pathways with periodic updates to remain in lockstep with changing technology	Literature calls for integrating AI into nursing, but prospective evidence linking specific competencies to patient and health system outcomes remains limited.
5. Define and Monitor Performance Metrics	Adopt a minimum monitoring guide for each model (clinical performance, equity, workload, drift).	Establish metrics: (1) discrimination + calibration, (2) subgroup performance (race/ethnicity, language, sex, age, payer, SDOH), (3) workload & safety (alert burden, overrides, near-misses, reported harms), (4) drift (data/performance checks with escalation thresholds). Create dashboards for real-time decision-making; use findings for updates/retraining, and governance adjustments.	At implementation and as part of routine quality review (eg, 30/90-day gates, quarterly reviews, summaries to governance and service lines	The AICC calls for monitoring but does not specify cadence/thresholds. Lessons from high-profile cases (Epic Sepsis Model^15^; Obermeyer et al^16^) should be used to justify concrete gates and equity segmentation.

Abbreviations: AI, artificial intelligence; AICC, Artificial Intelligence Code of Conduct; CE, continuing education; EHR, electronic health record; IT, information technology; NAM, National Academy of Medicine; RACI, Responsible, Accountable, Consulted, Informed; SDOH, social determinant of health.

Source: author’s analysis.

Operationalizing nursing participation in AI governance will require structural and resource support—such as protected time for nurse leaders to serve on governance committees, funding for AI literacy and competency training, and data access mechanisms that enable nurses to review model inputs and outputs. Health systems can phase this capacity building by integrating nursing representation into existing quality and safety governance structures, supported by continuing education and dedicated informatics roles.

## Conclusion: a shared path forward for nursing and health systems in implementing the AICC

The AICC framework introduced by the NAM marks a significant milestone for ethical healthcare innovation. It highlights safety, transparency, equity, accountability, and active participation of the healthcare workforce but does not specify the roles and responsibilities of particular clinicians, such as nurses, in AI governance. The AICC’s core principles including equity, beneficence, transparency, and accountability resonate with the American Nurses Association’s Code of Ethics. Aligning the 2 frameworks underscores that responsible AI governance is inherently aligned with nursing values and professional obligations. Responsible AI development and implementation require not only following ethical principles but also a fundamental change in how health systems and the nursing workforce approach technology integration. The AICC provides a guiding framework that can explicitly include nursing perspectives to ensure that AI tools positively impact healthcare delivery and improve the quality and equity of care for everyone. Ongoing and foundational education for nurses and frontline clinicians is essential to promote the responsible use of AI in rapidly evolving health systems.

While this commentary emphasizes nurses as a key underrepresented group, effective AI governance also requires engagement of other stakeholders to achieve holistic governance and trustworthy AI integration. Beyond nursing, effective AI governance should also include perspectives from pharmacists, social workers, case managers, and patient and family representatives to ensure that governance processes represent all who are affected by AI in healthcare. Finally, advancing nursing inclusion in AI governance requires targeted funding for interdisciplinary research, academic–industry partnerships, and policy mechanisms that formally embed nursing representation at governance tables. These actions will sustain equity, accountability, and workforce trust across the AI lifecycle.

## Data Availability

No data were generated in this study.
